# The impact of antibiotic use on outcomes of relapsed/refractory multiple myeloma patients treated with CAR-T therapy

**DOI:** 10.3389/fimmu.2025.1566016

**Published:** 2025-04-17

**Authors:** Lingling Yin, Bin Lv, Jiao Ge, Yuekun Qi, Jieyun Xia, Sha Ma, Ying Wang, Yang Liu, Dian Zhou, Jiang Cao, Zhiling Yan, Kunming Qi, Wei Sang, Depeng Li, Hai Cheng, Wei Chen, Kailin Xu, Weiying Gu, Zhenyu Li, Feng Zhu

**Affiliations:** ^1^ Blood Diseases Institute, Xuzhou Medical University, Xuzhou, Jiangsu, China; ^2^ Department of Hematology, The Affiliated Hospital of Xuzhou Medical University, Xuzhou, Jiangsu, China; ^3^ Jiangsu Key Laboratory of Bone Marrow Stem Cells, Xuzhou, Jiangsu, China; ^4^ Department of Hematology, The First People’s Hospital of Changzhou, The Third Affiliated Hospital of Suzhou University, Changzhou, Jiangsu, China

**Keywords:** chimeric antigen receptor T cell, antibiotic, relapsed/refractory, multiple myeloma, outcome

## Abstract

**Background:**

In recent years, chimeric antigen receptor (CAR)-T cell therapy has achieved tremendous efficacy in relapsed/refractory multiple myeloma (R/R MM). However, the impact of antibiotic (ATB) use on R/R MM patients treated with CAR-T is still not known. The aim of our study was to analyse the influence of ATB on the clinical outcomes of R/R MM patients treated with CAR-T cells.

**Methods:**

In this retrospective study, 199 patients with R/R MM who received CAR-T cells between January 2018 and December 2023 were evaluated from two hospitals in China. They were stratified into ATB-group and No ATB-group according to whether ATB was administered in the 4 weeks before therapy. We mainly analyzed the efficacy, survival outcomes and cytotoxicity of CAR-T cell therapy in two groups of patients.

**Result:**

In the ATB group (90 patients), the overall response rate (ORR) was 70% comparable to the No ATB group (109 patients: ORR, 81.7%; *P* = 0.054). The complete response rate (CRR) was 40%, which was significantly lower compared with No ATB group (CRR, 57.8%; *P* = 0.012). The median progression-free survival (PFS) was 6.7 months while the median overall survival (OS) was 21.9 months for the ATB group. The median PFS and OS for the No ATB group were 13.9 months and 36.1 months. There were significant differences in PFS (*P* = 0.007) and OS (*P* = 0.004) between the evaluated groups. Nonetheless, multivariate analysis found ATB use did not reduce the CRR (odds ratio [OR], 0.947; 95% confidence interval [CI], 0.251 to 3.565, *P* = 0.936). Besides, administration of ATB did not affect the PFS (hazard ratio [HR], 0.634; 95% CI, 0.28 to 1.436, *P* = 0.275) and OS (HR, 2.259; 95% CI, 0.755 to 6.762, *P* = 0.145) in R/R MM patients treated with CAR-T cells. Additionally, both groups of patients had similar incidences of cytokine release syndrome (CRS) and immune effector cell-associated neurotoxicity syndrome (ICANS).

**Conclusion:**

Our results point to a detrimental effect of ATB on treatment outcomes to CAR-T cell therapy. However, the use of ATB is not associated with the incidence of CRS or ICANS.

## Introduction

Chimeric antigen receptor (CAR)-T cell therapy represents a breakthrough in the treatment of relapsed/refractory multiple myeloma (R/R MM). Especially in recent years, it has shown unprecedented antitumor efficacy and stands at the novel forefront of current R/R MM therapy (1–3). Numerous studies have shown that the R/R MM patients treated with anti-B cell maturation antigen (anti-BCMA) CAR-T cells have overall response rates (ORR) ranging from 81% to 100% (4–8). The combination of anti-BCMA and anti-CD19 CAR-T cells has shown a manageable long-term safety profile, with an ORR as high as 92% and durable responses (9). G protein-coupled receptor, class C group 5 member D (GPRC5D), another promising target, has also demonstrated extremely high safety and efficacy (10–13). More recently, our center reported that anti-BCMA/GPRC5D bispecific CAR-T cells yielded 86% ORR and 62% complete response rate (CRR) with no fatal adverse events (14). However, despite the favorable outcomes, not all R/R MM patients respond to CAR-T therapy (15). Patients who do not respond to CAR-T treatment often experience disease progression and may even suffer severe CAR-T mediated adverse effects, such as cytokine release syndrome (CRS) or immune effector cell-associated neurotoxicity syndrome (ICANS) (16). Thus, the search for potential factors influencing its efficacy is urgent and extremely necessary for a more targeted selection of treatment populations in clinical practice.

Gut microbiota is increasingly considered an important factor associated with both tumor development and the effect of T cell-driven anticancer immunotherapy (17). Recently, growing evidence has indicated that gut microbiota signatures may be harnessed to predict therapeutic response or adverse effects in optimizing CAR-T cell therapy (18–21). Hu et al. observed significant differences in diversity and abundance of *Bifidobacterium*, *Prevotella*, *Sutterella*, and *Collinsella* between MM patients treated with second-generation BCMA CAR-T cells in complete remission (CR) and those in partial remission (PR) (21). In another recent study, Smith and colleagues found that higher abundance of *Ruminococcus, Bacteroides* and *Faecalibacterium* were associated with response to CD19 CAR-T cell therapy (22).

Antibiotic (ATB) therapy is commonly performed in clinic for R/R MM patients, who are more susceptible to infection because of hypogammaglobulinemia or treatment-related immune suppression. ATBs are potentially life-saving medicines, but they can impair the homeostasis of gut microbiota, resulting in decreased microbial abundance and diversity. Therefore, it is necessary to determine whether ATB use affects the efficacy of CAR-T treatment and the prognosis of R/R MM patients. Nowdays, the association between ATB use and the prognosis of cancer in CAR-T cell therapy remains controversial. Uribe-Herranz et al. found that mice receiving vancomycin in combination with CD19-directed CAR-T cell therapy showed increased tumor response and tumor-associated antigens (TAAs) cross-presentation compared with those of mice receiving CD19 CAR-T cell therapy alone in lymphoma murine model (23). Smith et al. conducted the first human study to investigate the influence of ATBs on the response and toxicity of anti-CD19 CAR-T cell therapy in patients with B cell malignancies. They demonstrated that exposure to ATBs in general and in particular to broad-spectrum anaerobe-targeting ones (piperacillin/tazobactam, meropenem and imipenem/cilastatin) within 4 weeks before therapy was associated with worse survival and increased neurotoxicity (22). In another recent study by Stein-Thoeringer et al., an association between exposure to ATBs prior to CD19 CAR-T cell infusion and increased incidences of cancer relapse/disease progression and worse overall survival (OS) in lymphoma patients was also observed (24). However, the impact of ATB on CAR-T cell efficacy in R/R MM has not been established.

Thus, in order to learn about the specific association between ATB use and CAR-T treatment of R/R MM patients and provide potential reference to clinic performance, we carried out a retrospective analysis to investigate the impact of ATB on outcomes of CAR-T cell therapy in R/R MM.

## Methods

### Patient selection

A retrospective cohort study was performed on R/R MM patients who received CAR-T cells at the Affiliated Hospital of Xuzhou Medical University and The First People’s Hospital of Changzhou during the period from January 2018 to December 2023 (ChiCTR1900026219, ChiCTR2000033194, ChiCTR2100048888). Patients age 18-75 years with good performance status (Karnofsky Performance score ≥50), a life expectancy of more than 12 weeks and adequate organ function. Patients with active infections, psychological or mental illnesses, severe allergies, or a history of severe allergies were excluded. The detailed inclusion and exclusion criteria could refer to our previous studies (10, 25). All patients were infused with BCMA- or GPRC5D-directed CAR-T cells. CAR structures were as described previously (10, 26, 27). Lymphodepletion regimen was cyclophosphamide (750 mg/m^2^/d, day -5) and fludarabine (30 mg/m^2^/d, days -5 to -3). All patients were followed-up until death or data lock (October 2024). The study was conducted with the approval from the ethics committee of the Affiliated Hospital of Xuzhou Medical University and The First People’s Hospital of Changzhou. Written informed consent was obtained from all patients in accordance with the Declaration of Helsinki.

### Clinical data collection

Clinicopathologic characteristics of patients were collected at enrollment, including age, sex, MM type, International Staging System (ISS) stage, Karnofsky Performance score (KPS), tumor burden, prior treatment, cytogenetic abnormalities and extramedullary diseases (EMD). The class and indication of ATB treatment 4 weeks before CAR-T cell infusion were collected, if available. In order to avoid the influence of antiviral drugs on the results, patients with COVID-19 and other viral infections were not included in our study. Laboratory data was obtained by retrieving electronic medical records. Peripheral blood T cell counts (CD3, CD4, CD8) were assessed at leukapheresis. The levels of c-reactive protein (CRP), lactate dehydrogenase (LDH), beta-2 microglobulin (β2-MG), and erythrocyte sedimentation rate (ESR) were recorded at lymphodepletion.

### Assessment of clinical outcomes

The International Myeloma Working Group criteria was used to evaluate clinical response of patients with R/R MM (28). Progression-free survival (PFS) was calculated from CAR-T cell infusion to the date of disease progression or death. OS was defined as the time from CAR-T cell infusion to death from any cause. CRS or ICANS was graded according to the American Society for Transplantation and Cellular Therapy consensus grading system (29).

### Statistical analysis

Descriptive statistics included medians (ranges) for all continuous variables and numbers (percentages) for categorical variables. The continuous variables were analyzed by the Mann-Whitney U test. χ^2^ or the Fisher’s exact test were used for categorical variables. Logistic regression was performed to estimate risk factors of CRR after CAR-T cell infusion. PFS and OS survival curves were estimated by the Kaplan-Meier method. Cox proportional hazards regression models were used for the analysis of clinical factors related to survival. Two-tailed *P* < 0.05 was considered statistically significant. All statistical analyses were performed using the IBM SPSS 27.0 software.

## Results

### Patient characteristics

**Figure 1:**
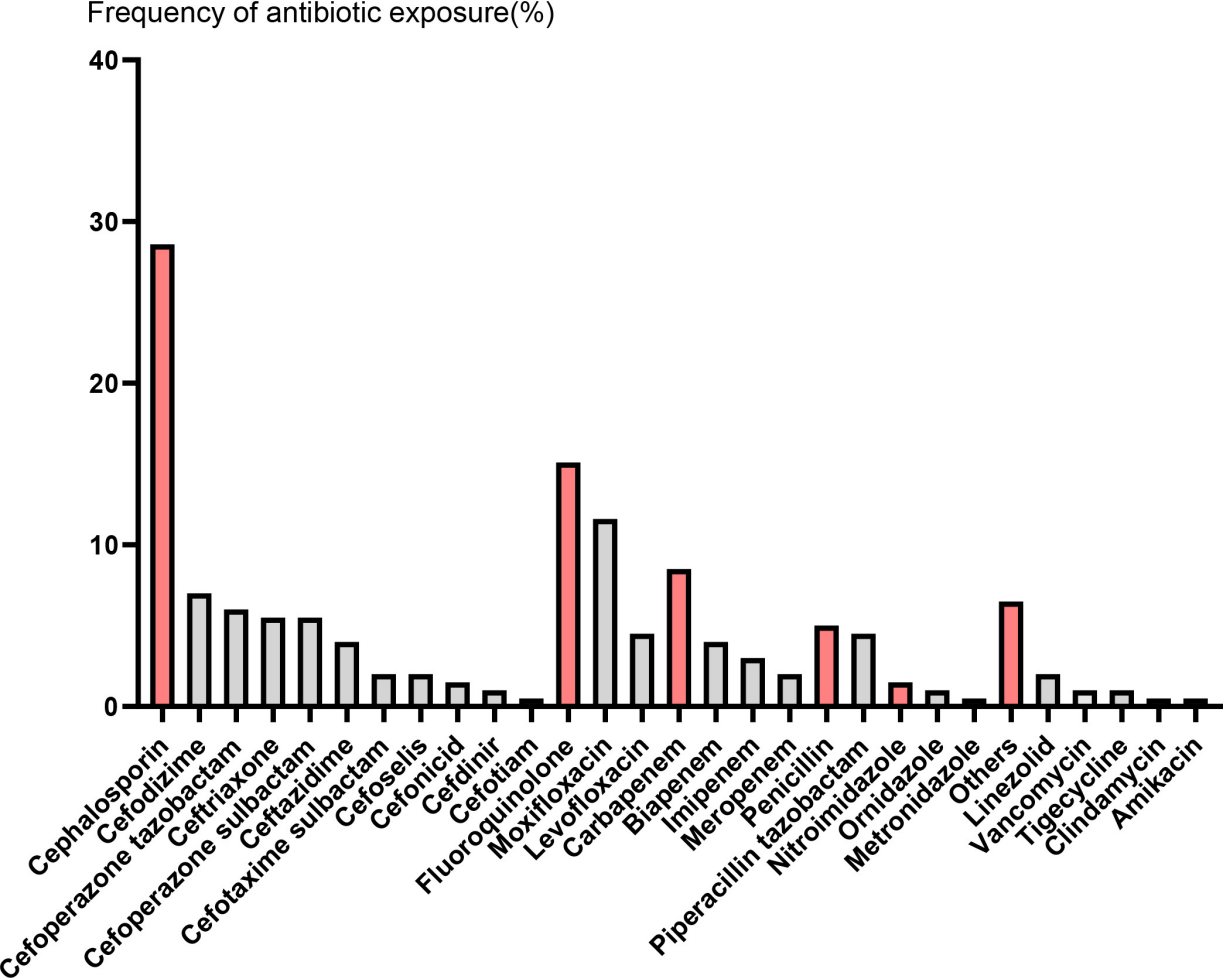
The frequency of antibiotic exposure in the four weeks prior to CAR-T cell infusion. Red columns represent different types of antibiotics, followed by gray columns that represent specific antibiotic names.

We identified 199 R/R MM patients treated with CAR-T cell during the study period. The median age was 59 years (range 29-74), and 53.8% of patients were male. Amongst this cohort, 90 (45.2%) patients received ATB within 4 weeks before CAR-T cell infusion and 109 (54.8%) patients did not. Respiratory tract infections were the most common indication for ATB prescriptions. By far, cephalosporin (CEP) was the most frequently administered ATB (63.3% of exposed patients), followed by 33.3% fluoroquinolone (FLU) (**Figure 1**, **Table 1**). The clinical characteristics of the patients are shown in **Table 2**. There was statistical difference between the two groups in terms of KPS (*P* = 0.033) and previous therapy lines (*P* = 0.014). Compared to the No ATB group, patients in the ATB group more frequently received a prior auto-hematopoietic stem cell transplantation (41.1% *vs*. 24.8%, *P* = 0.014). In the ATB group, the proportion of patients with EMD was significantly higher than that in the No ATB group (*P* = 0.002). Other baseline characteristics were not remarkably different between the two groups.

**Table 1:** Classes and indications for antibiotic (ATB) in patients receiving CAR-T cell therapy.

		Number(%^†^)
ATB Class*	Cephalosporin	57(63.3)
	Fluoroquinolone	30(33.3)
	Carbapenem	17(18.9)
	Penicillin	10(11.1)
	Nitroimidazole	3(3.3)
	Linezolid	4(4.4)
	Vancomycin	2(2.2)
	Tigecycline	2(2.2)
	Clindamycin Amikacin	1(1.1) 1(1.1)
Indication for ATB treatment	Respiratory Tract Infection	81(90.0)
	Gastrointestinal Infection	3(3.3)
	Urinary Tract Infection	2(2.2)
	Skin Infection	1(1.1)
	Unclear source	3(3.3)

*Some patients received multiple classes so there is overlap between categories.

†% of exposed patients.

**Table 2:** Baseline characteristics and association with ATB use.

	Total	ATB	No ATB	*P*
No.(%)	199	90(45.2)	109(54.8)	
Male,no.(%)	107(53.8)	45(50.0)	62(56.9)	0.333
Age,median(range)	59 (29-74)	60(32-73)	58(29-74)	0.297
KPS,no.(%)				0.033
50-60	22(11.1)	14(15.6)	8(7.3)	
70-80	21(10.6)	13(14.4)	8(7.3)	
90-100	156(78.4)	63(70.0)	93(85.3)	
Types,no.(%)				0.772
IgG	106(53.3)	45(50.0)	61(56.0)	
IgA	38(19.1)	19(21.1)	19(17.4)	
IgD	16(8.0)	6(6.7)	10(9.2)	
Light chain	34(17.1)	17(18.9)	17(15.6)	
Non-secretory	5(2.5)	3(3.3)	2(1.8)	
ISS,stage III,no.(%)	63(31.7)	29(32.2)	34(31.2)	0.8
*High tumor burden,no.(%)	21(10.6)	9(10.0)	12(11.0)	0.867
†High-risk cytogenetics,no.(%)	48(24.1)	24(26.7)	24(22.0)	0.416
Previous auto-HSCT,no.(%)	64(32.2)	37(41.1)	27(24.8)	0.014
Previous therapy lines	4(1-17)	4(1-16)	4(1-17)	0.014
‡EMD,no.(%)	66(33.2)	40(44.4)	26(23.9)	0.002
Target of CAR-T cells,no.(%)				0.806
BCMA	128(64.3)	56(43.8)	72(56.2)	
GPRC5D	53(26.3)	26(49.1)	27(50.9)	
BCMA+GPRC5D	18(14.1)	8(44.4)	10(55.6)	

*High tumor burden was defined as at least 50% plasma cells in bone marrow.

†High-risk cytogenetics: presence of del(17p), t(4;14), t(14;16), and amp(1q).

‡Extramedullary disease (EMD) included tissue masses in extraosseous locations and bone-related plasmacytomas.

KPS, karnofsky performance score; ISS, International Staging System; Auto-HSCT, auto-hematopoietic stem cell transplantation.

Two-sided *P* values were calculated using the Mann–Whitney U test for continuous variables and χ^2^ or Fisher’s exact test for categorical variables.

### ATB and survival outcomes

**Figure 2:**
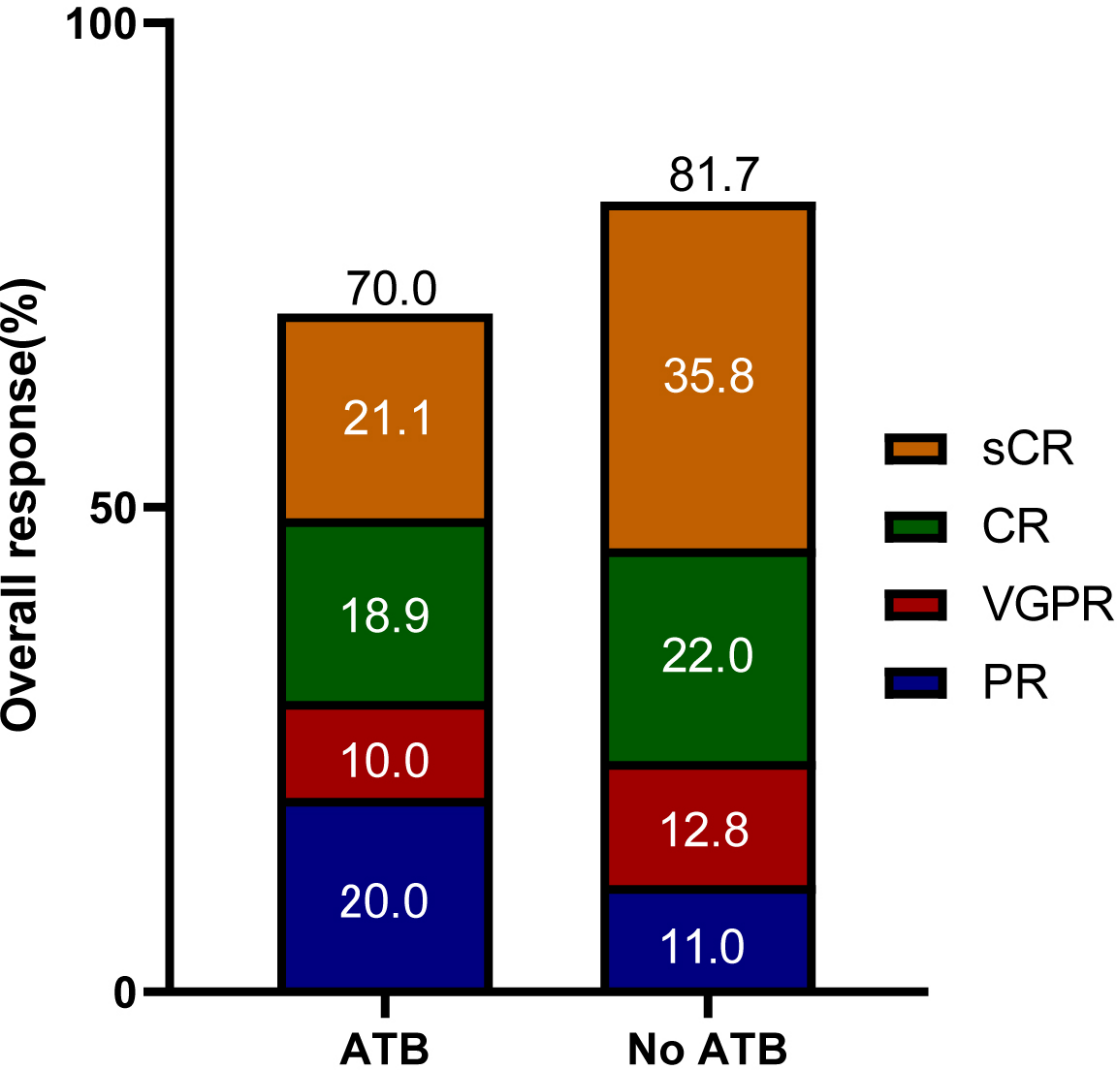
Overall responses of patients in the ATB group and No ATB group. sCR, stringent complete response; CR, complete response; PR, partial response; VGPR, very good partial response.

Patients achieved the best response within a median time of 1.8 months (range 0.4-7.3). In the ATB group, ORR was 70.0%, including 19 (21.1%) stringent complete response (sCR), 17 (18.9%) CR, 9 (10.0%) very good partial response (VGPR), and 18 (20.0%) PR. In the No ATB group, ORR was 81.7%, including 39 (35.8%) sCR, 24 (22.0%) CR, 14 (12.8%) VGPR, and 12 (11.0%) PR (**Figure 2**). The ues of ATB did not affect the ORR (*P* = 0.054). In contrast, difference in CRR between the ATB group and No ATB group was significant (*P* = 0.012) (**Table 3**). Owing to the varied effects of different ATB on the gut microbiota (30), we investigated whether different ATB could affect the efficacy of CAR-T therapy differently. We analyzed CEP and FLU for this purpose, which were selected as they were the most commonly used among patients, with CEP prescribed to 57 patients (28.6% of all patients) and FLU to 30 patients (15.1%), making them suitable for statistical analysis (**Figure 1**). Patients who received CEP had lower ORR (*P* = 0.005) and CRR (*P* = 0.009) compared to patients not receiving CEP. Likewise, patients received FLU had lower CRR (*P* = 0.002) than those who did not (**Table 3**).

**Table 3:** Association between antibiotic and responses.

	ATB use		*P*	CEP use		*P*	FLU use		*P*
	ATB (n=90)	No ATB(n=109)		Yes(n=57)	No(n=142)		Yes(n=30)	No(n=169)	
Overall response,no.(%)	63(70.0)	89(81.7)	0.054	36(63.2)	116(81.7)	0.005	22(73.3)	130(76.9)	0.67
Best overall response,no.(%)									
Complete response or better	36(40.0)	63(57.8)	0.012	20(35.1)	79(55.6)	0.009	7(23.3)	92(54.4)	0.002
Complete response	17(18.9)	24(22.0)		8(14.0)	33(23.2)		3(10.0)	38(22.5)	
Stringent complete response	19(21.1)	39(35.8)		12(21.1)	46(32.4)		4(13.3)	54(32.0)	
Very good partial response	9(10.0)	14(12.8)		8(14.0)	15(10.6)		3(10.0)	20(11.8)	
Partial response	18(20.0)	12(11.0)		8(14.0)	22(15.5)		12(40.0)	18(10.7)	

Response status was determined by the International Myeloma Working Group criteria. ATB is considered for any antibiotic exposure. Yes indicates the use of cephalosporin (CEP) or fluoroquinolone (FLU). *P* values were calculated with χ^2^ test.

Considering the impact of ATB on CRR, we queried whether patients who were exposed to ATB were the ones with more advanced disease and disease-related comorbidities that led to ATB therapy. We further analyzed the clinical factors that might be associated with efficacy of CAR-T cell therapy in patients with R/R MM. Univariate analysis confirmed that ATB therapy was associated with poor response to CAR-T treatment but lost its association in multivariate analysis (**Table 4**). Multivariate analyses stratified by the types of ATB showed that CEP and FLU were not independent factors affecting CRR. In contrast, high-risk cytogenetics was an independent predictive factor for better CRR.

Table 4Clinical factors associated with complete response rate (CRR) in patients with R/R MM.

**Table:** 

A CRR of patients exposed to ATB						
Variable	CR	non-CR	Univariable		Multivariable	
	(n=99)	(n=100)	*P*	OR	95% CI	*P*
Male,no.(%)	53(53.5)	54(54.0)	0.948			
Age,median(range)	57(32-71)	60(29-74)	0.058	0.95	0.862-1.048	0.307
KPS,no.(%)			0.003			0.275
50-60	5(5.1)	17(17.0)		0.388	0.053-2.868	
70-80	6(6.1)	15(15.0)		0.254	0.038-1.672	
90-100	88(88.9)	68(68.0)		(Reference)		
ISS,stage III,no.(%)	29(29.3)	34(34.0)	0.537			
High tumor burden,no.(%)	4(4.0)	17(17.0)	0.007	0.826	0.133-5.109	0.837
High-risk cytogenetics,no.(%)	16(16.2)	32(32.0)	0.065	0.247	0.062-0.992	0.049
Previous auto-HSCT,no.(%)	29(29.3)	35(35.0)	0.389			
Previous therapy lines	4(1-15)	4(1-17)	0.047	0.824	0.597-1.138	0.24
EMD,no.(%)	25(25.3)	41(41.0)	0.019	1.19	0.303-4.666	0.803
CRP,median(range)	2.05(0-152)	3.9(0-229)	0.127			
LDH,median(range)	193(29-736)	188.5(89-1813)	0.232			
β2-MG,median(range)	2682.5(782-10240)	3343(1008-17296)	0.028	1.000	1.000-1.000	0.188
ATB,no.(%)	36(36.4)	54(54.0)	0.013	0.947	0.251-3.565	0.936

**Table:** 

B CRR of patients exposed to CEP				C CRR of patients exposed to FLU			
Variable	Multivariable			Variable	Multivariable		
	OR	95% CI	*P*		OR	95% CI	*P*
Age	0.951	0.864-1.048	0.312	Age	0.949	0.86-1.047	0.293
KPS			0.288	KPS			0.172
50-60	0.396	0.054-2.905		50-60	0.3	0.038-2.381	
70-80	0.26	0.039-1.716		70-80	0.192	0.028-1.336	
90-100	(Reference)			90-100	(Reference)		
High tumor burden	0.764	0.128-4.552	0.767	High tumor burden	0.635	0.1-4.005	0.632
High-risk cytogenetics	0.25	0.063-0.995	0.049	High-risk cytogenetics	0.218	0.053-0.898	0.035
Previous therapy lines	0.826	0.595-1.147	0.254	Previous therapy lines	0.855	0.631-1.158	0.312
EMD	1.15	0.287-4.614	0.843	EMD	1.115	0.278-4.467	0.878
β2-MG	1.000	1.000-1.000	0.232	β2-MG	1.000	1.000-1.000	0.186
CEP	0.576	0.156-2.131	0.409	FLU	0.236	0.029-1.932	0.178

**(A)** Univariable and multivariable analyses of CRR in patients who were exposed to ATB in the four weeks before CAR-T cell therapy. **(B, C)** Multivariable analysis of CRR in patients who were exposed to CEP or FLU.

CRP, c-reactive protein; LDH, lactate dehydrogenase; β2-MG, beta-2 microglobulin.

Logistic regression was performed to estimate clinical factors associated with CRR. The statical threshold for inclusion of a variable in the multivariate model was <0.10.

**Figure 3:**
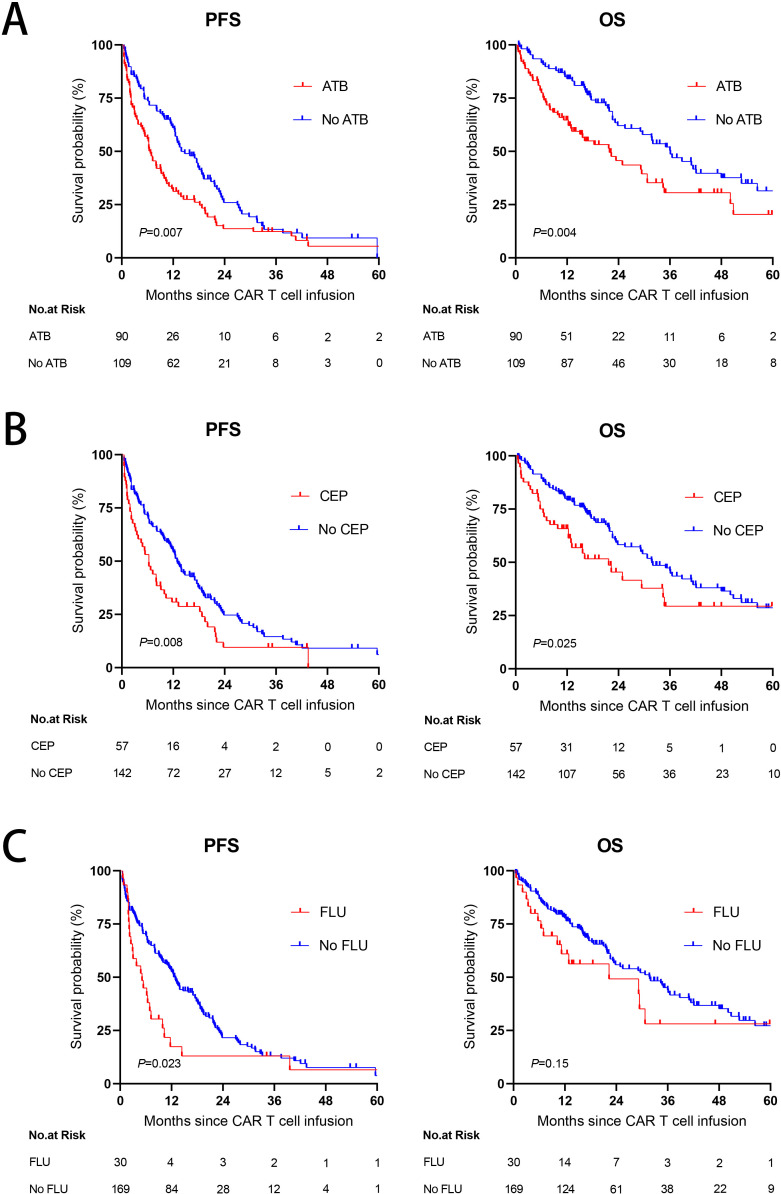
The impact of antibiotic use on PFS and OS. **(A)** Kaplan-Meier survival curves in R/R MM populations according to exposure to ATB within the 4 weeks before CAR-T cell infusion. **(B, C)** Kaplan-Meier curves of PFS and OS according to exposure to CEP **(B)** or FLU **(C)** within the 4 weeks before CAR-T cell infusion. *P* values were shown (log-rank test).

We next examined whether the use of ATB affected the PFS or OS in these patients. At a median follow-up of 16.9 months, the median PFS was only 6.7 months (95% CI, 5.055 to 8.345) and the median OS was only 21.9 months (95% CI, 13.664 to 30.136) in the ATB group, which were significantly shorter than those in the No ATB group (median PFS: 13.9 months, 95% CI, 9.63 to 18.17; median OS: 36.1 months, 95% CI, 27.226 to 44.974) (**Figure 3A**). Since CEP and FLU were commonly used, we also explored their effects on CAR-T cell immunotherapy long-term outcomes. Exposure to CEP during the 4 weeks preceding CAR-T cell infusion was associated with worse PFS (*P* = 0.008) and OS (*P* = 0.025) (**Figure 3B**). PFS was lower in patients treated with FLU (*P* = 0.023), with a trend toward a decreased rate in OS (*P* = 0.15) (**Figure 3C**).

**Figure 4:**
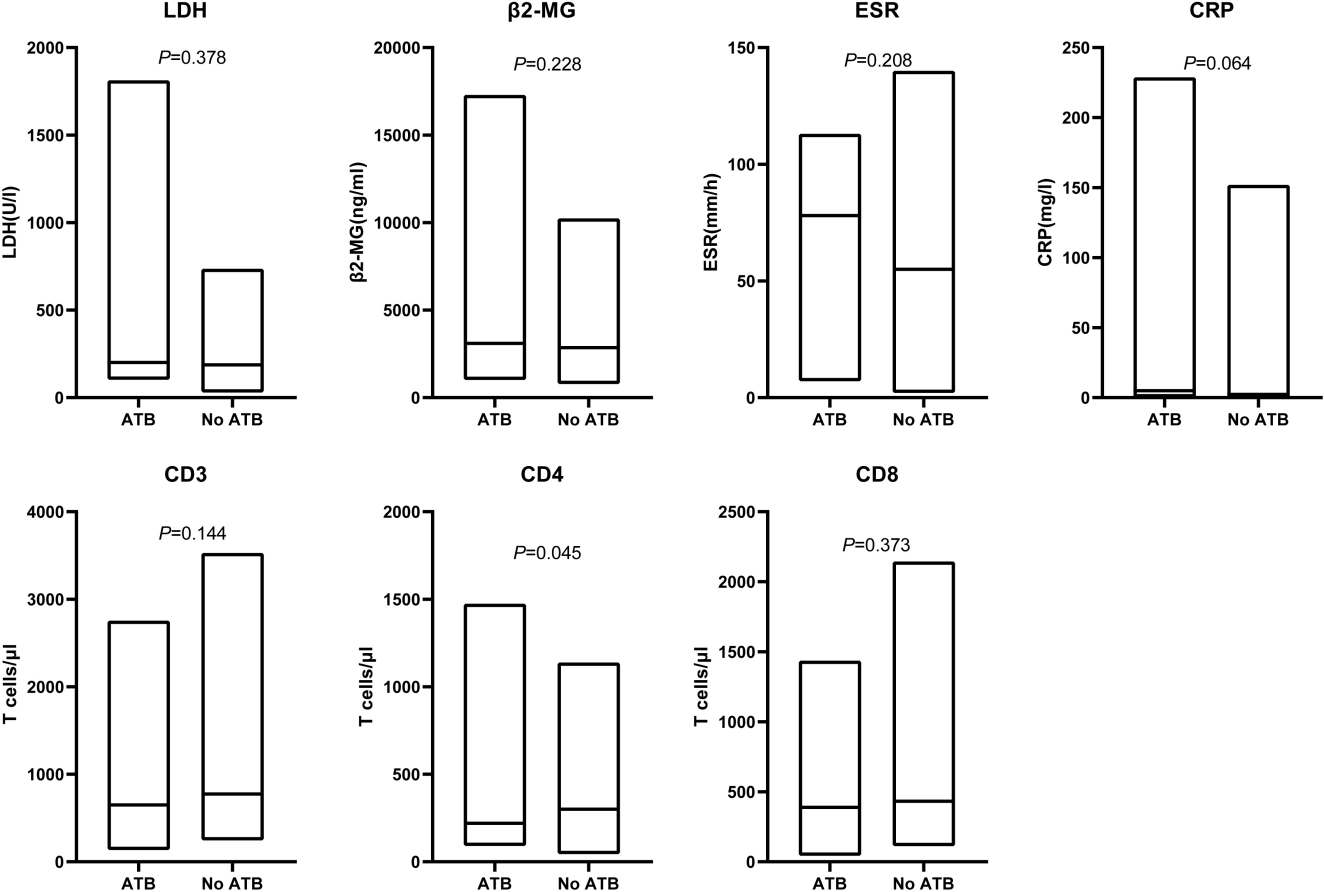
Associations of ATB use and tumor burden, systemic inflammation, peripheral blood T cell counts. Serum levels of LDH, β2-MG, ESR, CRP before lymphodepletion. Peripheral blood CD3, CD4 and CD8 T cell counts at the time point of leukapheresis. LDH, lactate dehydrogenase; β2-MG, beta-2 microglobulin; ESR, erythrocyte sedimentation rate;CRP, c-reactive protein. Line within the box plots indicates median, lower box bound indicates min, upper box bound indicates max. *P* values were calculated with the Mann–Whitney U test.

ATB-treated patients could feature more aggressive disease and/or deteriorated clinical status which may have a potential confounding effect on survival. To evaluate these possibilities, we investigated tumor burden and systemic inflammation in our patients prior to CAR-T cell therapy initiation. LDH or β2-MG as a surrogate marker for tumor burden had similar level between the ATB group and No ATB group (LDH: *P* = 0.378; β2-MG: *P* = 0.228). To eliminate the confounding effect of baseline inflammatory status on survival, we investigated differences in baseline inflammatory markers such as ESR and CRP. No associations were observed between ATB administration and ESR (*P* = 0.208), CRP (*P* = 0.064). In addition to tumor burden and physical status, the quality of autologous CAR-T cells also affects survival outcomes, which is heavily dependent on the quality of the T cells harvested from the patient. Intriguingly, patients receiving ATB displayed significantly lower peripheral CD4 T cell counts (*P* = 0.045) and a trend towards lower CD3 (*P* = 0.144) and CD8 T cells (*P* = 0.373) (**Figure 4**).

Finally, to further determine whether ATB was an independent prognostic factor for PFS and OS, we carried out univariate and multivariate analyses of the effect of ATB administration, taking into account classical prognostic factors relevant to R/R MM. The univariate analysis revealed that the use of any ATB or CEP was a risk factor affecting the PFS (ATB: HR 1.53, 95% CI, 1.121 to 2.087, *P* = 0.007; CEP: HR 1.571, 95% CI, 1.118 to 2.206, *P* = 0.009) and OS (ATB: HR 1.762, 95% CI, 1.197 to 2.595, *P* = 0.004; CEP: HR 1.617, 95% CI, 1.057 to 2.474, *P* = 0.027). However, multivariate analysis confirmed that ATB or CEP utilization had no independent adverse influence on PFS or OS. Univariate and multivariate analyses found that FLU also significantly affected the PFS (HR 4.185, 95% CI, 1.285 to 13.631, *P* = 0.018) of the patients, with no statistical significance in OS (HR 1.321, 95% CI, 0.334 to 5.227, *P* = 0.691) (**Table 5**).

Table 5Univariable and multivariable analyses of progression-free survival (PFS) and overall survival (OS).

**Table:** 

A PFS and OS according to the use of ATB				
Variable	Univariable		Multivariable	
	HR(95% CI)	*P*	HR(95% CI)	*P*
PFS				
Sex, male	1.345(0.986-1.834)	0.061	1.294(0.635-2.636)	0.478
Age	0.99(0.97-1.01)	0.305		
KPS		0.001		0.424
50-60	2.523(1.542-4.127)		0.998(0.268-3.721)	
70-80	1.18(0.714-1.948)		2.005(0.704-5.71)	
90-100	(Reference)		(Reference)	
ISS,stage III	0.906(0.644-1.274)	0.569		
High tumor burden	2.13(1.295-3.502)	0.003	6.492(2.202-19.136)	<0.001
High-risk cytogenetics	1.249(0.742-2.103)	0.403		
Previous auto-HSCT	1.93(1.371-2.718)	<0.001	2.798(1.225-6.393)	0.015
Previous therapy lines	1.056(1.007-1.107)	0.025	0.904(0.785-1.041)	0.161
EMD	2.281(1.645-3.163)	<0.001	0.89(0.405-1.958)	0.773
CRP	1.006(1.001-1.011)	0.019	1.01(0.999-1.022)	0.075
LDH	1.002(1.002-1.003)	<0.001	1.005(1.002-1.008)	<0.001
β2-MG	1.000(1.000-1.000)	0.005	1.000(1.000-1.000)	0.031
ATB	1.53(1.121-2.087)	0.007	0.634(0.28-1.436)	0.275
CEP	1.571(1.118-2.206)	0.009		
FLU	1.6(1.031-2.482)	0.025		
OS				
Sex, male	1.386(1.01-2.187)	0.044	1.032(0.386-2.757)	0.95
Age	0.997(0.972-1.023)	0.826		
KPS		<0.001		0.01
50-60	4.588(2.705-7.781)		4.048(0.599-27.367)	
70-80	1.254(0.68-2.311)		6.4(1.82-22.503)	
90-100	(Reference)		(Reference)	
ISS,stage III	0.933(0.616-1.412)	0.742		
High tumor burden	2.723(1.576-4.706)	<0.001	3.626(1.063-12.376)	0.04
High-risk cytogenetics	1.227(0.654-2.304)	0.524		
Previous auto-HSCT	2.304(1.513-3.51)	<0.001	1.638(0.539-4.979)	0.384
Previous therapy lines	1.067(1.007-1.13)	0.028	1.005(0.845-1.197)	0.951
EMD	2.875(1.939-4.263)	<0.001	0.683(0.231-2.024)	0.492
CRP	1.007(1.001-1.013)	0.016	1.007(0.994-1.02)	0.3
LDH	1.001(1.001-1.002)	0.002	1.003(0.999-1.007)	0.088
β2-MG	1.000(1.000-1.000)	0.048	1.000(1.000-1.000)	0.683
ATB	1.762(1.197-2.595)	0.004	2.259(0.755-6.762)	0.145
CEP	1.617(1.057-2.474)	0.027		
FLU	1.394(0.805-2.414)	0.154

**Table:** 

B PFS and OS according to the use of CEP						
Variable	Multivariable(PFS)			Multivariable(OS)		
	HR	95% CI	*P*	HR	95% CI	*P*
Sex, male	1.263	0.611-2.608	0.528	1.07	0.394-2.91	0.894
KPS			0.487			0.013
50-60	0.976	0.251-3.794		4.682	0.729-30.062	
70-80	1.921	0.654-5.637		5.797	1.708-19.68	
90-100	(Reference)			(Reference)		
High tumor burden	5.927	2.041-17.216	0.001	3.802	1.139-12.689	0.03
Previous auto-HSCT	2.299	1.09-4.849	0.029	2.389	0.93-6.135	0.07
Previous therapy lines	0.905	0.782-1.048	0.183	0.999	0.844-1.182	0.986
EMD	0.796	0.362-1.751	0.571	0.824	0.27-2.518	0.734
CRP	1.009	0.998-1.02	0.112	1.01	0.997-1.022	0.13
LDH	1.005	1.002-1.008	<0.001	1.003	0.999-1.007	0.91
β2-MG	1.000	1.000-1.000	0.043	1.000	1.000-1.000	0.808
CEP	1.263	0.611-2.608	0.528	1.509	0.563-4.043	0.413

**Table:** 

C PFS and OS according to the use of FLU						
Variable	Multivariable(PFS)			Multivariable(OS)		
	HR	95% CI	*P*	HR	95% CI	*P*
Sex, male	0.971	0.47-2.006	0.937	1.118	0.405-3.084	0.829
KPS			0.358			0.016
50-60	1.385	0.371-5.172		4.406	0.677-28.679	
70-80	2.24	0.722-6.949		5.539	1.64-18.705	
90-100	(Reference)			(Reference)		
High tumor burden	5.943	2.1-16.82	<0.001	4.014	1.199-13.437	0.024
Previous auto-HSCT	2.131	0.994-4.568	0.052	2.655	1.068-6.6	0.036
Previous therapy lines	0.909	0.782-1.057	0.215	0.995	0.841-1.178	0.958
EMD	1.026	0.463-2.272	0.95	0.771	0.248-2.39	0.652
CRP	1.003	0.992-1.013	0.605	1.009	0.995-1.023	0.196
LDH	1.006	1.003-1.009	<0.001	1.003	1.000-1.007	0.08
β2-MG	1.000	1.000-1.000	0.286	1.000	1.000-1.000	0.832
FLU	4.185	1.285-13.631	0.018	1.321	0.334-5.227	0.691

**(A)** Univariable and multivariable analyses of the association of ATB exposure prior to CAR-T cell infusion and PFS or OS. **(B, C)** Multivariable analysis of CEP or FLU exposure and PFS or OS.

The statical threshold for inclusion of a variable in the multivariate model was <0.10. *P*-value for interaction calculated with Cox proportional hazards model.

### ATB and CRS or ICANS

**Figure 5:**
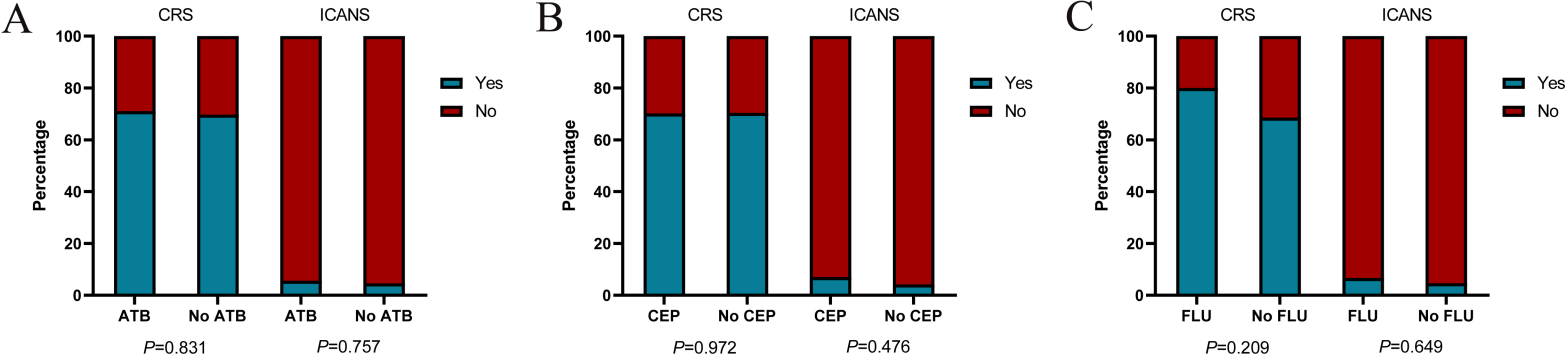
The incidences of cytokine release syndrome (CRS) and immune effector cell-associated neurotoxicity syndrome (ICANS) in patients with CAR-T cell therapy. Histograms show the frequencies of CRS and ICANS according to exposure to any ATB **(A)**, CEP **(B)**, FLU **(C)** within the four weeks before CAR-T cell infusion. Blue (Yes) indicates the presence of CRS or ICANS of any grade, while red (No) indicates the absence of CRS or ICANS of any grade. *P* values were calculated using χ^2^ or Fisher’s exact test.

Of all patients, 70.4% experienced CRS. Grade 3 or higher CRS, defined as severe CRS, occurred in 6 (3%) patients. Median time to onset of CRS was 8d (0-29 d), median duration of CRS was 4d (1-25d). In the ATB group, 36 (40%) patients had grade 1, 24 (26.7%) had grade 2, and 4 (4.4%) had grade≥ 3 CRS. In the No ATB group, 49 (45%) patients had grade 1, 25 (22.9%) had grade 2, and 2 (1.8%) had grade 3 CRS. ICANS occurred in 10 (5%) of 199 patients, and 4 (2%) patients had grade ≥3 ICANS. Median time to onset of ICANS was 10d (1-26 d), median duration of ICANS was 4d (1-13d). In the ATB group, 3 (3.3%) patients had grade 1, 1 (1.1%) had grade 2, and 1 (1.1%) had grade 4 ICANS. In the No ATB group, 2 (1.8%) patients had grade 1 and 3 (2.8%) had grade 3 ICANS. No significant difference was found in the incidence of CRS (*P* = 0.831) or ICANS (*P* = 0.757) between the two groups (**Figure 5A**). Similarly, we did stratified analyses of ATB classes, and the results were consistent (**Figures 5B, C**).

## Discussion

With the increased use of CAR-T in cancer therapeutics, tremendous effort has been made to search possible factors that influence its efficacy. Among the identified factors, increasing evidence has indicated a crucial role for the gut microbiota (18, 31). It is well known that ATB is the most common clinical cause of alterations in gut flora. There are theoretical concerns about whether the use of ATB will impair the efficiency of CAR-T cells. Currently, limited studies exploring the impact of ATB on clinical outcomes of CAR-T cell therapy are focused on lymphoma and acute lymphoblastic leukemia (ALL) (22, 24, 32), and no studies on MM have been conducted. To the best of our knowledge, this is the first retrospective study to investigate the relationship between ATB and CAR-T efficacy in the treatment of R/R MM.

In the present study, R/R MM patients who received ATB prior to CAR-T cell therapy had poor treatment response and worse long-term survival. Our results align with the previous cancer immunotherapy literature (33–37). In one study, patients with stage III and IV melanoma exposed to ATBs before initiation of immune checkpoint inhibitors (ICI) had statistically significantly worse OS than unexposed patients (38). Moreover, ATB exposure was associated with greater moderate to severe immune-mediated colitis. Similarly, in a study of 251 patients, of whom the 135 who received ATBs had lower response rates and shorter survival. Further research found that ATB exposure was associated with changes in certain cytokines and antibodies. In the lung cancer patients, they observed differences in interferon-gamma (IFN-γ), interleukin-8 (IL-8), and macrophage inflammatory proteins (39). In sum, ATB use is associated with poor therapeutic outcomes of immunotherapy. Our study also proved this view in CAR-T as a new immunotherapy method. In addition, Eng et al. (40) observed that ATB exposure, especially of FLU, prior to ICI therapy correlated with reduced OS. Similarly, Pederzoli et al. (41) linked FLU use to an increased recurrence rate. We evaluated the effects of CEP and FLU, the most commonly used ATBs in clinical hematology at our hospital, on CAR-T efficacy, indicating that FLU significantly and adversely affected the PFS but not OS. It may be attributed to our small sample size, which needs more studies for further verification.

Establishing a causal relationship between ATB administration and poorer prognosis in patients undergoing CAR-T cell therapy is challenging. Two scenarios were deduced for the impact of ATB on CAR-T efficacy. In the first scenario, ATB use, even in the short term, causes a loss of intestinal microbial diversity called dysbiosis, which can persist for up to several months after the ATB treatment (42, 43). Therefore, ATB-induced perturbation of the balanced microbiome would adversely impact CAR-T therapy efficacy. In the second scenario, patients with very low immunity might be more prone to infections, may be more often in need of ATB therapy. More and more studies showed that normal immune system further eliminate the residual tumor cells by the anti-tumor immunological reaction. Furthermore, immune failure in MM is the important factor for disease progress. Therefore, these individuals might inherently struggle to benefit from CAR-T cell therapy, potentially leading to reduced PFS and OS. In addition, those with cumulative ATB use could experience immunosuppression due to severe infections, which adversely affects the efficacy of CAR-T treatment. Thus, ATB treatment might simply reflect poor physical conditions rendering it a surrogate marker of dismal outcomes, without any relationship to its effects on the gut microbiome. Multivariate analysis correcting for ATB-independent markers of poor outcomes (such as age, sex, KPS, high-risk cytogenetics, prior lines of treatment, tumor burden, LDH, previous auto-HSCT, EMD, CRP and β2-MG) showed that ATB use was not independently associated with worse outcomes, suggesting that ATB indeed may not be an independent prognostic factor. Of course, validation of these findings in larger clinical studies will be needed.

CRS and ICANS are common side effects of CAR-T cell therapy. Another finding of our study was that ATB-exposed patients experienced similar rates of CRS and ICANS. Our results differ from prior findings in lymphoma (22, 24). It could be related to the profoundly different nature of MM compared to lymphoma. MM is characterized by an aggressive proliferation with the abnormal plasma cells being localized mostly in the bone marrow, while lymphoma cells are usually found in lymphoid organs. Secondly, the incidence of ICANS is relatively low due to the small sample size.

Notably, some inherent limitations do exist in our study. To begin with, this was a retrospective study, so unavoidable bias, confounding, and missing data would be anticipated. ATB use was entirely based on the patient’s electronic medical record and may not represent an accurate prescription of what the patient was currently taking. Conversely, the patient may have been prescribed but never taken antibiotic. In addition, the total number of patients analyzed was relatively small, which prevented us from conducting subgroup analyses based on the time of antibiotic used, route of administration, etc. Finally, we did not elucidate the mechanism by which ATB exert a detrimental effect on clinical outcomes. We speculate that ATB use causes dynamic changes in the gut microbiota that affect CAR-T efficacy.

An important future direction that would address our study’s limitations would be larger, multicenter studies with standardized prospective data collection. Further investigations should include the analysis of fecal microbiome so as to explore the mechanism of ATB affecting CAR-T treatment outcomes.

## Conclusion

In conclusion, the use of ATB prior to CAR-T therapy affects clinical outcomes of R/R MM patients. Given the known overutilization of ATB in current society, clinicians should exercise caution in patients who are scheduled to receive CAR-T therapy.

## Data Availability

The raw data supporting the conclusions of this article will be made available by the authors, without undue reservation.
